# Detection and genomic characterization of *Klebsiella pneumoniae* and *Escherichia coli* harboring *tet*(X4) in black kites (*Milvus migrans*) in Pakistan

**DOI:** 10.1038/s41598-024-59201-5

**Published:** 2024-04-20

**Authors:** Muhammad Hassan Mansoor, Xiaoyu Lu, Hanna Woksepp, Amna Sattar, Farwa Humak, Jabir Ali, Ruichao Li, Jonas Bonnedahl, Mashkoor Mohsin

**Affiliations:** 1 Institute of Microbiology, University of Agriculture, Faisalabad, 38000 Pakistan; 2 Jiangsu Co-Innovation Center for Prevention and Control of Important Animal Infectious Diseases and Zoonoses, College of Veterinary Medicine, Yangzhou University, Yangzhou, Jiangsu People’s Republic of China; 3 Department of Development and Public Health, Kalmar County Hospital, 391 85 Kalmar, Sweden; 4 Department of Chemistry and Biomedical Sciences, Linnaeus University, 392 31 Kalmar, Sweden; 5 Department of Biomedical and Clinical Sciences, Linköping University, 581 83 Linköping, Sweden; 6 Department of Infectious Diseases, Region Kalmar County, 391 85 Kalmar, Sweden

**Keywords:** *Escherichia coli*, *Klebsiella pneumoniae*, *tet*(X4), ICE*Kp2*, Birds, Antimicrobial resistance, Ecological epidemiology, Infectious diseases

## Abstract

The emergence of plasmid-mediated tigecycline resistance gene *tet*(X4) among clinically relevant bacteria has promoted significant concerns, as tigecycline is considered a last-resort drug against serious infections caused by multidrug-resistant bacteria. We herein focused on the isolation and molecular characterization of *tet*(X4)-positive *Klebsiella pneumoniae (K. pneumoniae)* and *Escherichia coli (E. coli)* in wild bird populations with anthropogenic interaction in Faisalabad, Pakistan. A total of 150 birds including black kites (*Milvus migrans*) and house crows (*Corvus splendens*) were screened for the presence of tigecycline resistance *K. pneumoniae* and *E. coli.* We found two *K. pneumoniae* and one *E. coli* isolate carrying *tet*(X4) originating from black kites. A combination of short- and long-read sequencing strategies showed that *tet*(X4) was located on a broad host range IncFII plasmid family in *K. pneumoniae* isolates whereas on an IncFII-IncFIB hybrid plasmid in *E. coli*. We also found an integrative and conjugative element ICE*Kp2* in *K. pneumoniae* isolate KP8336. We demonstrate the first description of *tet*(X4) gene in the WHO critical-priority pathogen *K. pneumoniae* among wild birds. The convergence of *tet*(X4) and virulence associated ICE*Kp2* in a wild bird with known anthropogenic contact should be further investigated to evaluate the potential epidemiological implications. The potential risk of global transmission of *tet*(X4)-positive *K. pneumoniae* and *E. coli* warrant comprehensive evaluation and emphasizes the need for effective mitigation strategies to reduce anthropogenic-driven dissemination of AMR in the environment.

## Introduction

Antimicrobial resistance (AMR) is a growing global threat to human and animal health driven by the selective pressure of extensive antibiotic consumption in multiple sectors including community settings, hospitals, veterinary, agriculture and aquaculture^1^. The issue has been highlighted in a systematic analysis, where AMR was associated with an estimated 5 million deaths in 2019, with 1.3 million deaths directly attributable to bacterial AMR^2^. The situation is further aggravated by the widespread distribution of AMR genes in the environment and insufficient investment in the development of new antibiotics^3^. The emergence of clinically relevant AMR in the environment primarily stems from pollution resulting from human activities. Additionally, this phenomenon is influenced by intricate interactions, including the genetic exchange of resistance genes facilitated by selective mechanisms enhanced by pollutants such as biocides and antibiotics^4^. The presence of clinically relevant multidrug-resistant (MDR) bacteria in the environment is a growing concern, with wildlife, particularly wild birds, being viewed as important sentinels for AMR surveillance^5,6^. Although wild birds can acquire antimicrobial-resistant bacteria/genes, likely from foraging in anthropogenically impacted areas including both landfills and WWTPs^7–9^, the role of wild birds in the dissemination of clinically relevant AMR needs further investigation^10^. Several studies have suggested that wild birds could be competent vectors of AMR and potentially disperse AMR through their movements^8,11,12^. Tigecycline is regarded as the last resort antibiotic in the clinical management of infections associated with MDR bacteria, particularly carbapenem and colistin-resistant *Enterobacteriaceae.* The recently discovered *tet*(X4) gene on plasmid confers resistance to tigecycline and has been found mainly in *E. coli* isolated from various sources, including humans, animals, and the environment^13^. The ability of the *tet*(X4) gene to be carried on various plasmid types, including hybrid plasmids, can facilitate its spread among bacterial populations and contribute to the emergence and spread of tigecycline-resistant bacteria.

Plasmids, such as IncFII, IncFIB, IncFIA, IncX1, IncQ1, IncA/C, IncHI1, and others, have been discovered to be the key carriers for propagating *tet*(X4)^14,15^. Tigecycline resistance in *K. pneumoniae* is mainly caused by mutations in the genes *ramR*, *soxR*, *oqxR*, *rpsJ*, and *tet*(A). However, the recent discovery of *tet*(X4) in *K. pneumoniae* is alarming, given *K. pneumoniae* is listed as a WHO critical-priority pathogen^16^.

Several studies have reported the presence of tigecycline-resistant *E. coli* carrying the *tet*(X4) gene in wild birds^17,18^, which raises concerns due to its potential spread in the environment. Additionally, this may lead to the transfer of *tet*(X4) to other bacteria, including those that cause human and animal infections. In this study, we investigated the prevalence and molecular characteristics of *tet*(X4) in *K. pneumoniae* and *E. coli* isolates from wild birds in Pakistan and further described the *tet*(X4)-harboring plasmids.

## Methods

### Sample collection and bacterial isolates

In this cross-sectional study, faecal droppings of 150 wild birds with known anthropogenic interaction (n = 100 from black kites and n = 50 from house crows) were collected from various public parks in Faisalabad, Pakistan during June 2022. A single fresh isolated faecal dropping sample was taken from an individual bird using sterile charcoal swabs and transported to the lab for microbiological examination. To prevent contamination, only the top surface of each dropping was swabbed, avoiding contact with the ground beneath. In addition, we confirmed the origin of the avian species by the direct visual observation of crows and kites in a public park. Collected samples were cultivated on Simmons citrate agar supplemented with amoxicillin and myo-inositol at concentrations of 10 µg/mL and 10%, respectively for the isolation of *Klebsiella* spp.^19^.

For the isolation of *E. coli,* UTI ChromoSelect agar (Merck, Darmstadt, Germany) was used. For the isolation of tigecycline-resistant colonies, sub-culturing of both *E. coli* and Klebsiella spp. was performed on UTI ChromoSelect agar supplemented with tigecycline (4 µg/mL). All the phenotypically resistant isolates were confirmed for species using API 20E biochemical strips (bioMérieux, Marcy l'Etoile, France). For the detection of *tet*(X4)-positive *E. coli* and *K. pneumoniae* isolates, PCR was performed using primers described earlier^20^. Briefly *tet*(X4)-gene was amplified using primer pairs tetX4-F (5ʹ-CCGATATTCATCATCCAGAGG-3ʹ) and tetX4-R (5ʹ CGCTTACTTTTCCAAGACTTACC-3ʹ) as forward and reverse primers with 32 cycles of denaturation at 95 °C for 30 s; annealing at 55 °C for 30 s; extension at 72° for 30 s^20^.

### Conjugation experiments

Conjugation assays were conducted to investigate the transferability of the *tet*(X4) positive isolates with sodium azide resistant *E. coli* J53 as the recipient strain. Transconjugants were selected on MacConkey agar containing (4 µg/mL) tigecycline combined with 100 µg/mL sodium azide. Subsequent carriage of *tet*(X4) bearing plasmids in the original parental strains and corresponding transconjugants was confirmed by PCR.

### Whole-genome sequencing and bioinformatics analysis

The total genomic DNA of isolates from overnight cultures was prepared using MagnaPure compact total NA kit according to the manufacturer’s instructions (Roche, Sweden). Library preparation was performed with the Illumina Nextera XT kit (Illumina, USA). Libraries were verified with the bioanalyzer high sensitivity DNA method (Agilent, USA). Paired-end sequencing (2 × 250 bp) of genomic DNA using a V3 run kit (Illumina) was performed on a MiSeq instrument (Illumina, San Diego, CA, United States). Short-read Illumina raw sequence reads were de novo assembled into contigs using SPAdes^21^ and contigs less than 500 bp were discarded. Analysis of multilocus sequence typing (MLST), acquired resistance genes, and plasmid replicons were conducted by the online tools MLST, ResFinder, and PlasmidFinder at the web service of Center for Genomic Epidemiology (http://www.genomicepidemiology.org/. Kleborate, which was designed specifically for *K. pneumoniae*, was used to identify virulence factors and ICE*Kp* structures, and further determine the sequence types (STs) of *K. pneumoniae*^22^.

To explore the evolutionary relationship of *tet*(X4)-positive *K. pneumoniae* between this study and other isolates in the NCBI database, all the *tet*(X4)-positive *K. pneumoniae* isolates were retrieved and downloaded from the NCBI Pathogen detection database. The isolates were retrieved with the search criteria ‘AMR_genotypes: *tet*(X4)’ and ‘Organism_group: *Klebsiella pneumoniae*’ as of March 2023. The draft assembled contigs were annotated using Prokka^23^, and then applied for phylogenetic analysis using Roary^24^ and FastTree^25^ based on single nucleotide polymorphisms (SNPs) of core genomes. The phylogenetic tree was visualized by iTOL (https://itol.embl.de/itol.cgi)^26^.

Genomic DNA was extracted using MagAttract HMW DNA kit according to the manufacturer’s instructions (Qiagen, Sweden) and was then subjected to long-read sequencing to obtain the complete sequences^27,28^. Library preparation and sample barcoding were performed using Rapid sequencing gDNA-barcoding chemistry and protocol (Oxford Nanopore Technologies, UK, SQK-RBK110.96, version RPK_9126_v110_revK_24Mar2021). Sequencing was performed using MinION™ MK-1B with FLO-MIN106 R9.4.1 flow cell and high-accuracy basecalling with read filtering out at Q score < 9 and trimming of barcodes, MinKnow 22.03.6 and Guppy 6.0.7. Short-read Illumina data and long-read Nanopore data were subjected to perform de novo assembly by Unicycler with the hybrid strategy. The rapid annotation website server (https://rast.nmpdr.org/rast.cgi) was then used to annotate the complete genome sequences^29^. Circular comparisons between *tet*(X4)-bearing plasmids and homologous plasmids available in the NCBI database were performed using the BRIG tool^30^. To visualize the genetic comparison features of ICE*Kp2*, Easyfig was used to generate linear comparison figures^31^.

## Results

### Identification of *tet*(X)-positive isolates and transferability

We identified two *K. pneumonia*e isolates (KP8333 and KP8336) and one *E. coli* isolate (EC8331) originating from black kites (*Milvus migrans*) with phenotypic resistance to tigecycline. All three isolates were also found to be positive for the carriage of *tet*(X4) gene and successfully transferred the *tet*(X4) gene into host *E. coli* J53 by conjugation.

### The phylogenetic analysis

**Figure 1:**
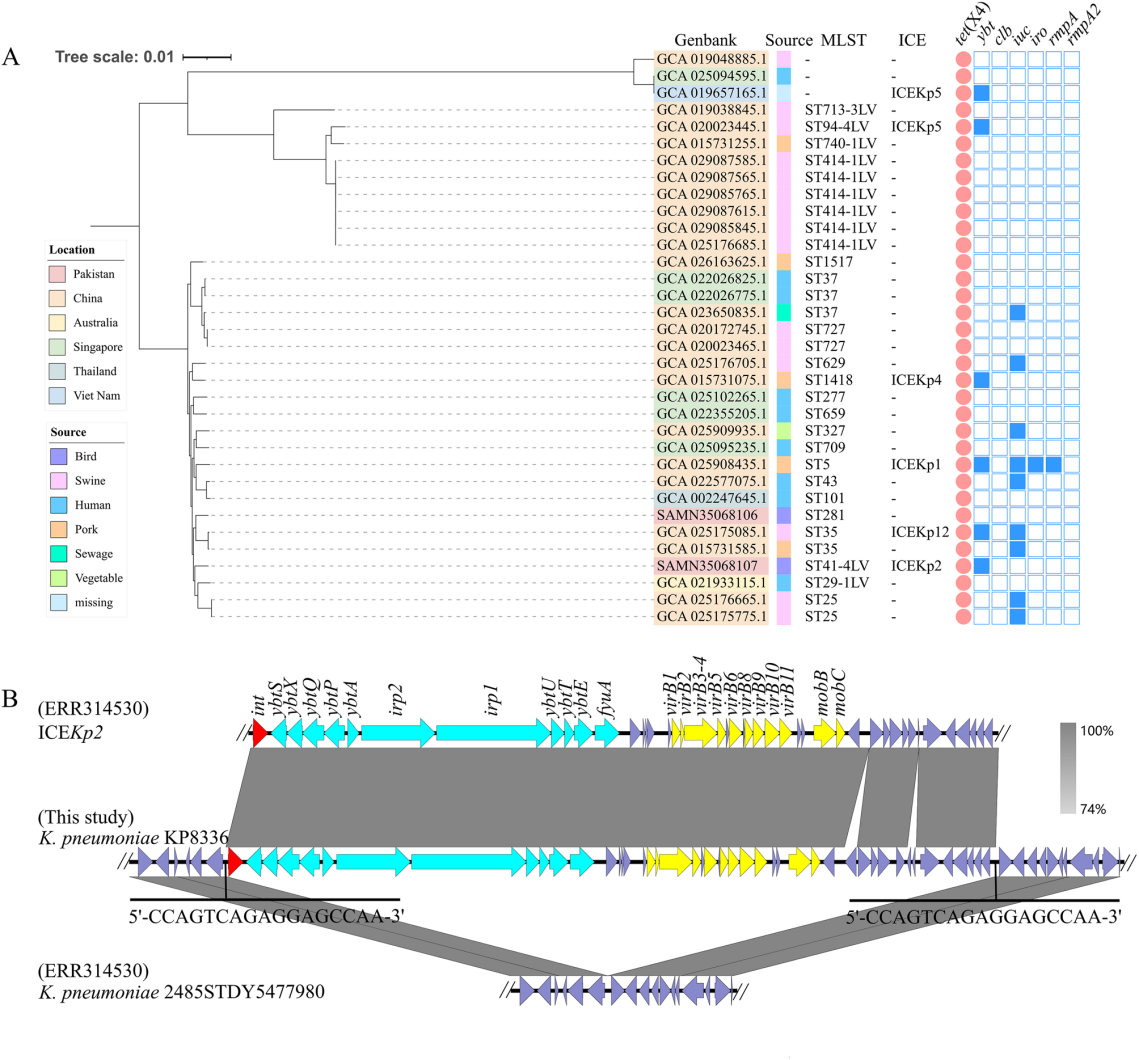
Phylogenetic tree of *tet*(X4)-positive *K. pneumoniae* strains and the ICE*Kp2* structure in KP8336. (**A**) Phylogenetic tree of 34 *tet*(X4)-positive *K. pneumoniae* strains, including two isolates in this study and 32 K*. pneumoniae* strains in the NCBI Pathogen detection database. The blue squares indicate the presence of virulence genes. (**B**) Alignment of the virulence-encoding region carried by KP8336, ICE*Kp2* structure in *K*. *pneumonia* 2485STDY5477980 and other chromosome regions of *K*. *pneumonia* 2485STDY5477980 (ERR314530).

To understand the evolutionary relationship of *tet*(X4)-carrying *K. pneumoniae* isolates, phylogenetic analysis of 34 *tet*(X4)-positive *K. pneumoniae* isolates (including two isolates in this study and 32 *K. pneumoniae* isolates in the NCBI Pathogen detection database) was performed. The phylogenetic analysis revealed that all 34 *K. pneumoniae* isolates were divided into two major clusters and none of the isolates displayed any clonal relationship with KP8333 or KP8336 (Fig. 1A). Among the *tet*(X4)-carrying *K. pneumoniae*, the majority was distributed in China (23/34), followed by Singapore (7/34). Only *K. pneumoniae* KP8333 and KP8336 in this study were from Pakistan. In addition, *tet*(X4)-carrying *K. pneumoniae* strains were prevalent in pigs (15/34), humans (9/34), and pork (5/34). The distribution of STs was diverse and only ST414-1LV *K. pneumoniae* strains from the pig in China demonstrate obvious clonal relationship (Fig. 1A). Virulence genes encoding yersiniabactin (*ybt*), colibactin (*clb*), aerobactin (*iuc*), salmochelin (*iro*), hypermucoidy (*rmp* and *rmpA2*) were detected whereas *clb* and *rmpA2* was not found in any *tet*(X4)-positive *K. pneumoniae* strains in this phylogenetic tree. Notably, five ICE*Kp* variants (ICE*Kp1*, ICE*Kp2*, ICE*Kp4,* ICE*Kp5*, and ICE*Kp12*) carrying *ybt* locus were found in six *K. pneumoniae* strains, and ICE*Kp2* appeared in KP8336.

### The ICE*Kp* structure in KP8336

The boundaries of ICE*Kp* in KP8336 was identified by the 17 bp direct repeats (5ʹ-CCAGTCAGAGGAGCCAA-3ʹ) formed upon integration (Fig. 1B). Comparative analysis indicated that the ICE*Kp* structure in KP8336 was one ICE*Kp2* variant, a 63 kb chromosomal island. The ICE*Kp2* structure mainly included the P4-like integrase gene *int*, the *ybt* locus (29 kb), and the sequence (14 kb) encoding the *virB*-type IV secretion system (T4SS) that is responsible for DNA transfer (Fig. 1B). In addition, the chromosome flanking ICE*Kp2* in KP8336 exhibited extremely high homology with the chromosome of *K. pneumoniae* 2485STDY5477980 isolated from human in UK (Fig. 1B).

### Genetic environment of *tet*(X4) in *E. coli*

**Figure 2:**
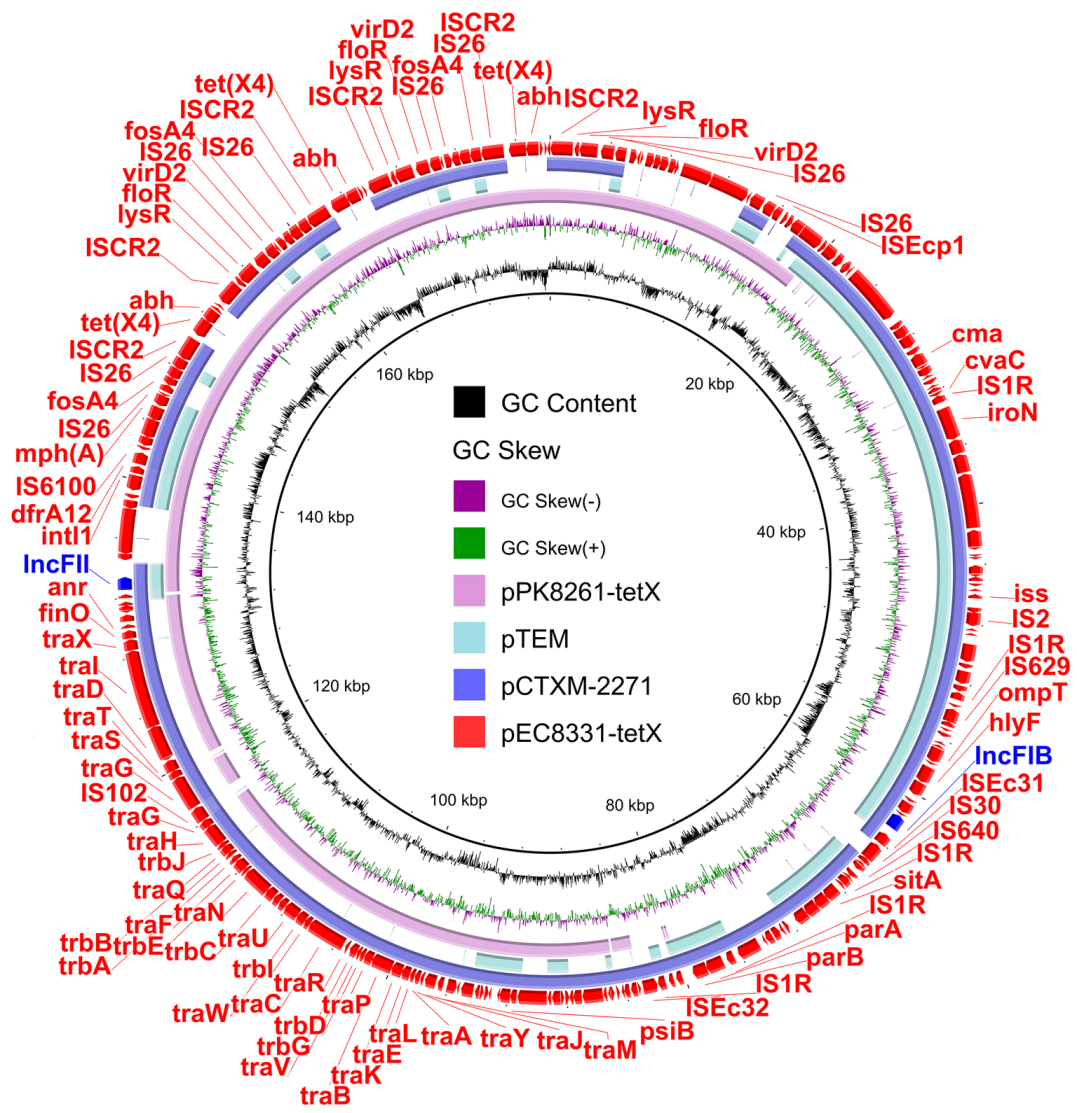
Circular comparison of *tet*(X4)*-*bearing plasmid in EC8331 and other similar plasmids in NCBI database. The pEC8331-tetX in this study were compared with plasmids pCTXM-2271, pTEM, and pPK8261-tetX in the NCBI database. The outermost circle indicates the plasmid pEC8331-tetX with genes annotated.

The isolate EC8331 was ST746 *E. coli* containing three plasmids: pEC8331-tetX, pEC8331-155 kb, and pEC8331-119 kb (Table 1). The *tet*(X4) gene was located on the IncFII-IncFIB (AP001918) plasmid pEC8331-tetX (178,255 bp), which is an MDR plasmid harboring *tet*(X4), *floR*, *fosA4*, *mph*(A), *dfrA12* and *bla*_TEM-215_ (Table 1 and Fig. 2). Sequence analysis revealed that pEC8331-tetX shared 99.85% identity at 86% coverage with IncFII-IncFIB (AP001918) plasmid pCTXM-2271 (MF589339) in *E. coli* 2271 from China (Fig. 2). The plasmid pEC8331-tetX also exhibited 99.99% identity at 43% coverage with IncFIB(AP001918) plasmid pTEM (CP047003) in *E. coli* J-8 from China, and 98.05% identity at 63% coverage with *tet*(X4)-bearing IncFII plasmid pPK8261-tetX (CP080156) in *E. coli* PK8261 isolated in chicken from Pakistan (Fig. 2). Evidently, it can be assumed that pEC8331-tetX was an evolved *tet*(X4)-positive IncFII-IncFIB (AP001918) hybrid plasmid and all antibiotic resistance genes on this plasmid were concentrated in the IncFII plasmid backbone structure region. The large repeat structure IS*26*-*hp*-*hp*-*fosA4*-*hp*-IS*26*-*virD2*-*floR*-*lysR*-IS*CR2*-*hp*-*abh*-*tet*(X4)-IS*CR2*-IS*26* with 13 kb in length was found in pEC8331-tetX (Fig. 2). The repeat structure was mediated by IS*26*, which has previously been reported to mediate tandem multiplication of genes within plasmids. In addition, *tet*(X4) was flanked by two copies of IS*CR2* with the structure IS*CR2*-*hp*-*abh*-*tet*(X4)-IS*CR2*.

**Table 1:** Characteristics of three *tet*(X4)-carrying isolates recovered from birds in Pakistan.

Strain IDs	Species	MLST	Components	Replicon types	Size (bp)	Resistance genes
EC8331	*E. coli*	ST746	EC8331-chromosome		4,685,870	None
			pEC8331-tetX	IncFII, IncFIB(AP001918)	178,255	*tet*(X4), *floR*, *fosA4*, *mph*(A), *dfrA12*, *bla*_TEM-215_
			pEC8331-155 kb	p0111	155,907	*aph(3'')-Ib*, *aph(6)-Id*, *tet*(A), *qnrS13*, *dfrA15*, *bla*_TEM-220_
			pEC8331-119 kb	IncI1-I(Alpha)	119,809	*aph(3')-Ia*, *aph(3'')-Ib*, *aph(6)-Id*, *bla*_TEM-126_, *dfrA15*, *qnrS13*, *tet*(A)
KP8333	*K. pneumoniae*	ST281	KP8333-chromosome		5,147,119	*fosA5*, *bla*_SHV-108_, *oqxA*, *oqxB*
			pKP8333-tetX	IncFII	100,799	*tet*(X4), *floR*, *fosA4*, *mph*(A), *dfrA12*, *bla*_TEM-215_
			pKP8333-201 kb	IncFIB(K)(pCAV1099-114)	201,997	None
			pKP8333-48 kb	IncFIA(HI1), repB(R1701)	48,790	*aph(6)-Id*, *aph(3'')-Ib*, *sul1*, *qacE*, *bla*_DHA-1_, *qnrB4*
			pKP8333-2 kb	ColRNAI	2563	none
KP8336	*K. pneumoniae*	ST41-4LV	KP8336-chromosome		5,456,436	*fosA5*, *bla*_SHV-108_, *oqxA*, *oqxB*
			pKP8336-tetX	IncFII(29)	66,571	*tet*(X4)
			pKP8336-167 kb	IncFIB(K), IncFII(K)	167,021	None

### Genetic environment of *tet*(X4) in *K. pneumoniae*

**Figure 3:**
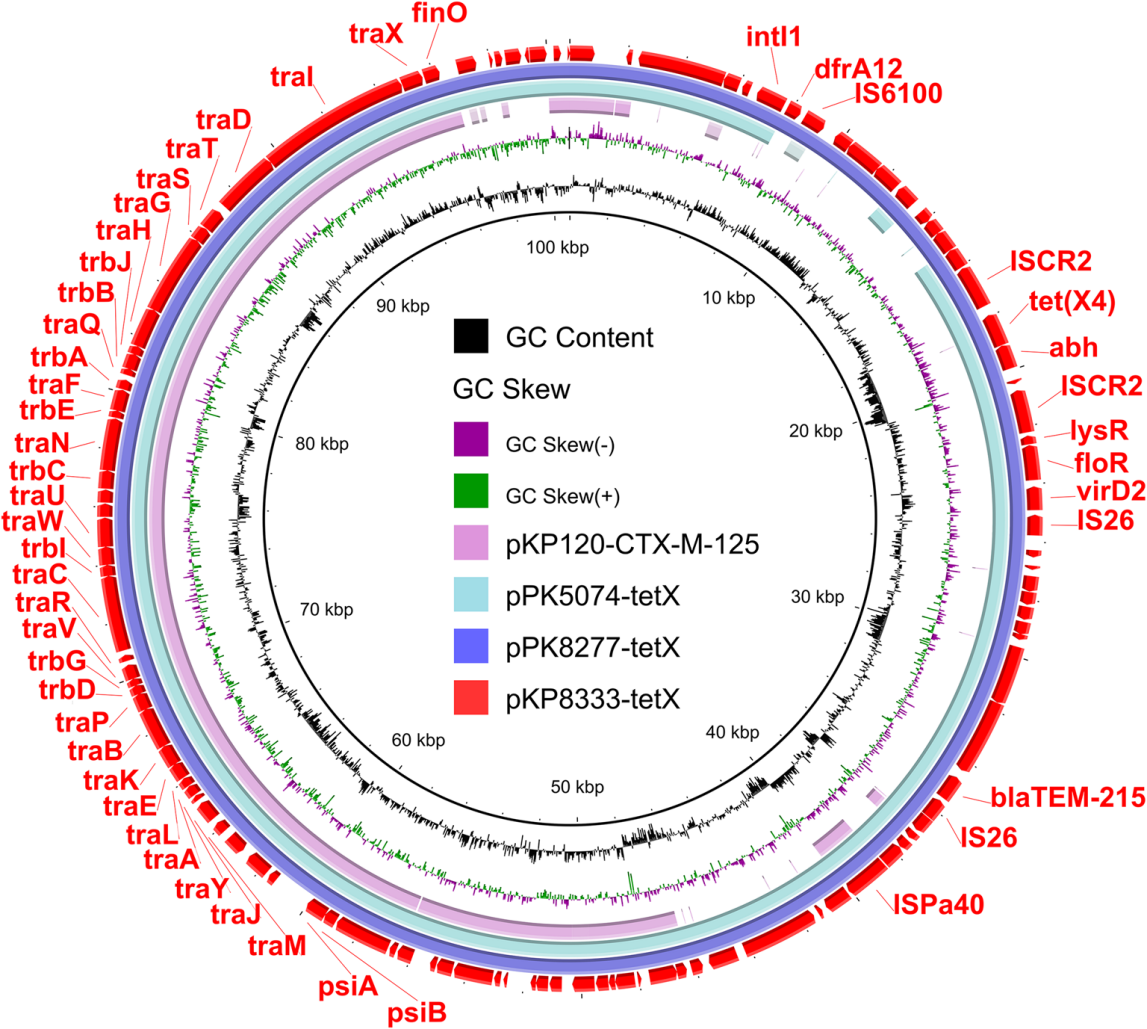
Circular comparison of *tet*(X4)*-*bearing plasmid in KP8333 and other similar plasmids in NCBI database. The pEC8331-tetX in this study were compared with plasmids pPK8277-tetX, pPK5074-tetX, and pKP120-CTX-M-125 in the NCBI database. The outermost circle indicates the plasmid pKP8333-tetX with genes annotated.

The *K. pneumoniae* isolate, KP8333, belonged to ST281 and harbored four plasmids: pKP8333-tetX, pKP8333-201 kb, pKP8333-48 kb, and pKP8333-2 kb (Table 1). The plasmid pKP8333-tetX was a *tet*(X4)-positive IncFII plasmid containing similar antibiotic resistance genes as pEC8331-tetX (Table 1 and Fig. 3). BLASTn search revealed that the plasmid pKP8333-tetX shared a high degree of genetic identity with the reported plasmids pPK8277-tetX (CP080134) (99.98% identity at 100% coverage) in chicken derived *E. coli* PK8277 and pPK5074-tetX (CP072807) (100.00% identity at 93% coverage) in human derived *E. coli* PK5074, which were from Pakistan (Fig. 3). Among *K. pneumoniae*, the plasmid pKP8333-tetX exhibited the highest similarity (97.77% identity at 55% coverage) to pKP120-CTX-M-125 (CP060746) (Fig. 3), suggesting that pKP8333-tetX is a newly emerging plasmid in *K. pneumoniae*.

**Figure 4:**
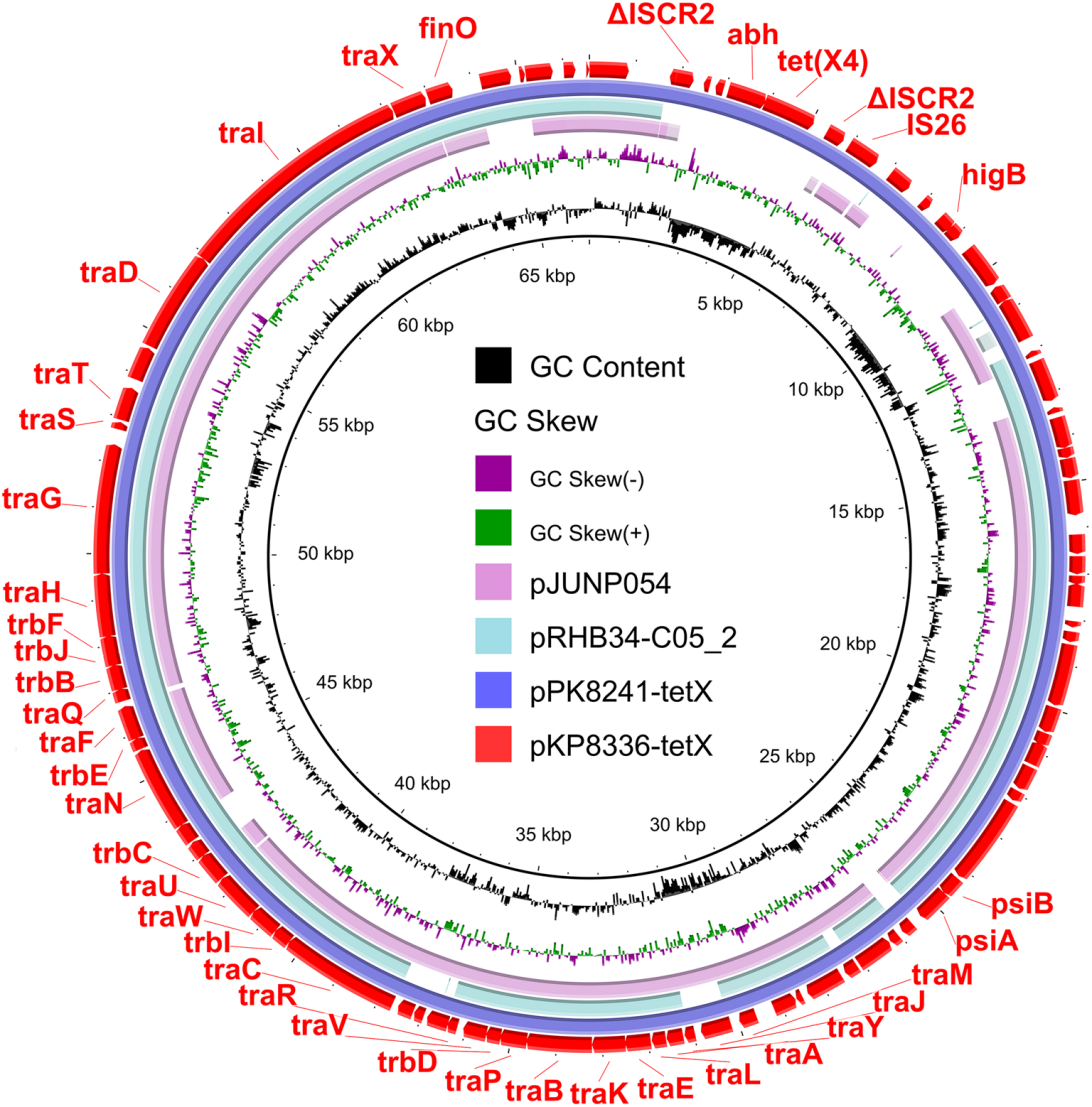
Circular comparison of *tet*(X4)*-*bearing plasmid in KP8336 and other similar plasmids in NCBI database. The pEC8331-tetX in this study were compared with plasmids pPK8241-tetX, pRHB34-C05_2, and pJUNP054 in the NCBI database. The outermost circle indicates the plasmid pKP8336-tetX with genes annotated.

The isolate KP8336 belonged to the ST41-4LV *K. pneumoniae*. Two plasmids pKP8336-tetX and pKP8336-167 kb were identified in KP8336 (Table 1). The pKP8336-tetX-66 kb was a *tet*(X4)-bearing IncFII(29) plasmid and no other resistance gene was found in this plasmid (Table 1 and Fig. 4). BLASTn analysis showed that pKP8336-tetX exhibited high homology (99.80% identity at 100% coverage) to the plasmid pPK8241-tetX (CP080140) in *E. coli* PK8241 from chicken in Pakistan (Fig. 4). The pKP8336-tetX also showed 94.96% identity at 83% coverage with plasmid pRHB34-C05_2 (CP057177) in *E. coli* RHB34-C05 and 94.96% identity at 83% coverage with plasmid pJUNP054 (LC506717) in *K. pneumoniae* JUNP054 (Fig. 4). This indicated that *E. coli* and *K. pneumoniae* are important host bacteria of *tet*(X4)-bearing IncFII(29) plasmids.

## Discussion

In this study, we isolated two *tet*(X4)-positive *K. pneumoniae* and one *tet*(X4)-positive *E. coli* isolate from black kites (*Milvus migrans*) in Faisalabad, Pakistan and performed a comparative genomic analysis on these isolates. Until now, only a few studies have reported the existence of *tet*(X4)-positive *K. pneumoniae* in human^16^, animal, and food specimens^32^ predominantly from China. This is the first report of the presence of *tet*(X4)-positive *K. pneumoniae* in Pakistan indicating the spread of *tet*(X4) beyond *E. coli*. Black kite is an abundant bird and an opportunistic and scavenging feeder providing ecosystem functions such as nutrient cycling and pest control etc. In Pakistan, black kites frequently visit anthropogenic affected areas such as landfills, food-producing animals and agricultural land^33^. One more indigenous reason for anthropogenic interaction is that a fraction of the population from a dominant religious group believes in giving pieces of flesh (cow/goat/chicken) as a means of warding off calamities and hardships^34^. Recent findings suggest a link between certain wild bird species and the acquisition of clinically relevant AMR. Notably, black kites have been reported to harbor carbapenem-resistant NDM-5-producing *E. coli*, in Pakistan^7^. Our finding of tet(X4)-positive *K. pneumoniae* in this wild bird species along with findings from previous studies is an indication that plasmid-mediated tigecycline resistance has the potential to disseminate within the one health framework like previously described with both ESBLs and carbapenem resistance genes^7,13,17,18,32,35^. Wild birds can acquire clinically relevant MDR bacteria, likely from foraging in anthropogenically impacted areas as reported earlier^36,37^. Tigecycline is rarely used in human medicine and is not used in food animals in Pakistan. However, plasmid-mediated *tet*(X4)-positive *E. coli* have been reported both from clinical and non-clinical settings^16,18^, which could be linked to excessive use of early generations of tetracycline antibiotics in food animals as proposed by several researchers^15,38^. However, the link between the long-term use of tetracyclines in food animals and the emergence of tigecycline resistance in bacteria needs to be ascertained.

Genome data found that both the *K. pneumoniae* isolates have different STs and showed no clonal relationship with the global *tet*(X4)-positive *K****.**** pneumoniae* strains in the NCBI database indicative of genetic diversity. Additionally, a single *tet*(X4)-positive *E. coli* isolate belonged to ST746 which has been previously associated with carbapenem resistance in Korea^39^ and China^40^. It has been suggested that mobile genetic elements, not the clones, play an important role in *tet*(X4) transmission^32^. We found an integrative and conjugative elements ICE*Kp2* in* K****.**** pneumoniae* KP8336 which carried *ybt* locus encoding yersiniabactin. It has been reported that ICE*Kp* variants could form an extrachromosomal circular intermediate and integrate into the chromosomes of recipient cells^41,42^. These results suggest that the formation of *tet*(X4)-positive hypervirulent *K. pneumoniae* KP8336 may be due to the horizontal transfer of ICE*Kp2* and IncFII(29) plasmid carrying *tet*(X4). The finding of ICE*Kp2* in *K. pneumoniae* from wild birds with known anthropogenic interaction is concerning. *K. pneumoniae* with ICE*Kp2* has been associated with clinical outbreaks^16^ and therefore, further investigative studies are important to be able to identify dissemination routes of AMR within the one health context. Additionally, mitigation efforts should be encouraged for already identified anthropogenic-driven AMR dissemination through waste, sewage and industrial pollution.

Hybrid genome assembly of *E. coli* EC8331 revealed that *tet*(X4) was located on a large MDR hybrid plasmid (~ 178 kb) IncFII-IncFIB (AP001918) plasmid harbouring additional resistance genes for quinolones, fosfomycin, macrolides, aminoglycosides and β-lactams. The hybrid plasmid pEC8331-tetX has large repeat structures surrounded by IS*26*, which has previously been reported to mediate tandem multiplication of genes within plasmids^43^. In addition, *tet*(X4) was flanked by two copies of IS*CR2,* forming a structure IS*CR2*-*hp*-*abh*-*tet*(X4)-IS*CR2*, which was found in several *tet*(X4)-positive isolates^20,44^. In previous studies from Pakistan, IncFII was the most common *tet*(X4) bearing plasmid ranging from 66 to 119 kbp in size^15,18^. *tet*(X4) bearing hybrid plasmids are being increasingly reported from China and are considered a new threat^41^. Therefore, the emergence of novel MDR hybrid plasmids in Pakistan is a serious concern because of their ability to contribute to the resistance and virulence genes co-translocation and demands continuous surveillance of AMR. Genomic comparison of *tet*(X4)-positive IncFII plasmid from *K. pneumoniae* KP8333 showed it has a similar genetic environment as that of *E. coli* isolated in this study and of chicken and human origins reported earlier in Pakistan^15,45^. These results indicate that *E. coli* and *K. pneumoniae* are important host bacteria of *tet*(X4)-bearing IncFII plasmids in Pakistan.

## Conclusion

The prevalence and molecular features of the *tet*(X4) positive bacteria in wild birds demonstrate that this gene has disseminated within the One Health framework and is yet an example of wild birds as potential carriers of novel plasmid-mediated resistance genes together with hypervirulent traits. This emphasizes the need for mitigation strategies for anthropogenic-driven dissemination of AMR in the environment.

As part of a comprehensive One Health strategy, we advocate for increased environmental AMR surveillance. To effectively monitor these efforts, we propose more detailed studies on wildlife and their interactions with known anthropogenic sources of AMR. By leveraging animal movement data from GPS telemetry, we can gain valuable insights into dynamics of AMR at the human–wildlife interface. Obvious mitigation strategies would be to expanding already existing interventions aimed at reducing AMR dissemination targeting landfills and wastewater treatment plants. Future strategies might also include reducing wildlife access to identified point sources of AMR.

A better understanding of the human–animal–wildlife interface will guide the development of evidence-based and effective One Health interventions that ultimately can reduce the AMR crisis.

## Data Availability

The datasets presented in this study can be found in online repositories. The names of the repository/repositories and accession number(s) can be found at DDBJ/ENA/GenBank under BioProject ID PRJNA972190. https://www.ncbi.nlm.nih.gov/bioproject/?term=PRJNA972190.
